# Cellular Structural Changes in *Candida albicans* Caused by the Hydroalcoholic Extract from *Sapindus saponaria* L.

**DOI:** 10.3390/molecules20059405

**Published:** 2015-05-22

**Authors:** Cristiane S. Shinobu-Mesquita, Patricia S. Bonfim-Mendonça, Amanda L. Moreira, Izabel C. P. Ferreira, Lucelia Donatti, Adriana Fiorini, Terezinha I. E. Svidzinski

**Affiliations:** 1 Departamento Análises Clínicas e Biomedicina, Universidade Estadual de Maringá, Av. Colombo 5790, bloco T20, Maringá 87020-900, Brazil; E-Mails: cristianeshinobu@gmail.com (C.S.S.-M.); patbonfim.09@gmail.com (P.S.B.-M.); 2 Departamento de Farmácia, Universidade Estadual de Maringá, Av. Colombo 5790, bloco K80, Maringá 87020-900, Brazil; E-Mails: amandalmoreira@gmail.com (A.L.M.); icpferreira@uem.br (I.C.P.F.); 3 Departamento de Biologia Celular, Universidade Federal do Paraná, Curitiba 85131-990, Brazil; E-Mail: donatti@ufpr.br; 4 Campus Palotina, Universidade Federal do Paraná, Palotina 85950-000, Brazil; E-Mail: drifiorini@gmail.com

**Keywords:** vulvovaginal candidiasis, *Sapindus saponaria*, antifungals, saponins

## Abstract

Vulvovaginal candidiasis (VVC) is a disease caused by the abnormal growth of yeast-like fungi in the mucosa of the female genital tract. *Candida albicans* is the principal etiological agent involved in VVC, but reports have shown an increase in the prevalence of *Candida* non-*C.* *albicans* (CNCA) cases, which complicates VVC treatment because CNCA does not respond well to antifungal therapy. Our group has reported the *in vitro* antifungal activity of extracts from *Sapindus saponaria* L. The present study used scanning electron microscopy and transmission electron microscopy to further evaluate the antifungal activity of hydroalcoholic extract from *S. saponaria* (HE) against yeast obtained from VVC and structural changes induced by HE. We observed the antifungal activity of HE against 125 vaginal yeasts that belonged to four different species of the *Candida* genus and *S. cerevisae*. The results suggest that saponins that are present in HE act on the cell wall or membrane of yeast at the first moments after contact, causing damage to these structures and cell lysis.

## 1. Introduction

Vulvovaginal candidiasis (VVC) is a disease caused by the abnormal growth of yeast-like fungi in the mucosa of the female genital tract. It afflicts millions of women worldwide annually, causing great discomfort, interfering with sexual and affective relations, and impairing work performance. VVC is classified by the World Health Organization as a frequently sexually transmitted disease, and it is considered an important world health issue [1].

*Candida albicans* is the principal etiological agent involved in VVC, but reports have shown an increase in the prevalence of *Candida* non-*C.* *albicans* (CNCA), mainly *C. glabrata*, in VVC [1,2,3]. In recent years, studies have reported decreases in the prevalence of vaginal *C. albicans* (e.g., by 65% [4], 68% [5], and 32.4% [6]). The increasing number of VVC cases caused by CNCA has complicated the diagnosis and treatment of VVC [7].

The therapeutic arsenal available for the treatment of fungal infections is quite restricted, limited to polyenic and azolic antifungal compounds [8,9]. For the treatment of VVC, nystatin (cream or vaginal ovule) has been used for decades, but therapeutic failure has been observed [10]. Azolic drugs inhibit the synthesis of ergosterol, an important component of the fungal cell membrane. Of the azolic drugs, fluconazole is the most used in VVC. However, it is expensive, and the development of resistance of *C. albicans* and CNCA yeasts to this drug has been reported [1]. These facts make the management of patients with VVC and recurrent VVC (RVVC) difficult and indicate the need to search for new, effective, safe, low-cost antifungal alternatives against this pathology [11]. The development of new antifungal agents, preferably from natural origins and with novel mechanisms of action, is an urgent medical need.

*Sapindus saponaria* L. (Sapindaceae) is a medium-sized deciduous tree found in the tropics, including America and India. Some authors have described the activity of extracts from *S. saponaria* against ulcers [12] and skin lesions caused by fungi [13] and antitumor and antimicrobial activity against Gram-positive bacteria, Gram-negative bacteria, and some filamentous fungi [14].

Our group has reported the antifungal activity of extracts from *S. saponaria* L. *in vitro* [15]. We found that hydroalcoholic and butanolic fractions of a crude extract from *S. saponaria* had *in vitro* and *in vivo* activity against azole-susceptible and -resistant human vaginal *Candida* species [16]. Still unknown, however, is the mechanism of action of hydroalcoholic fractions of extracts from *S. saponaria* [16] and damage to fungal cells by exposure to HE.

Then the aims of present study were evaluate the antifungal activity of HE against yeast from VVC and further to evaluate possible structural changes caused in *C. albicans* using scanning electron microscopy and transmission electron microscopy, with the goal of clarifying the possible mechanism of action of this fraction.

## 2. Results and Discussion

### 2.1. Preparation and Characterization of HE

**Figure 1:**
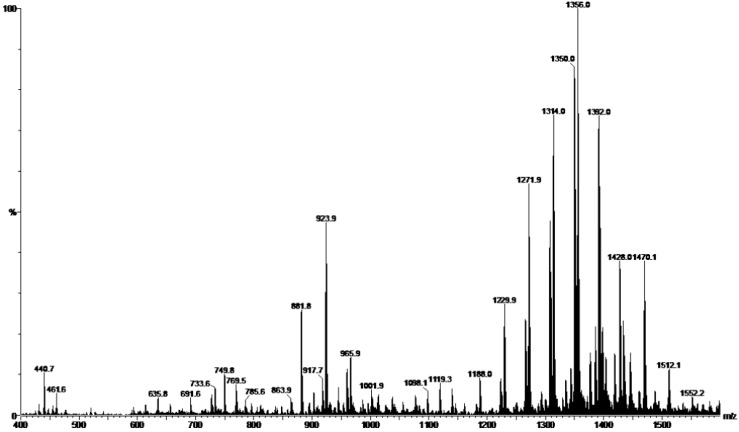
ESI-MS of HE obtained from fruits of *S. saponaria* L.

An hydroalcoholic fraction of the crude extract obtained from fruits of *S. saponaria* was directly injected into a mass spectrometer in negative ion mode, resulting in mass spectra that was identical to those reported by Tsuzuki [15] and too similar to obtained by Murgu and Rodrigues-Filho [13]. Based on these results, we were able to characterize this fraction of the extract and its chemical composition. Figure 1 shows the electron-spray ionization (ESI) mass spectra of the HE. Peaks were observed in the *m/z* 400–1550 region, corresponding to oligoglycoside acyclic sesquiterpene at *m/z* 1188–1512 and saponins at *m/z* 650–1000. The peak 881 represents a saponin deacetylated which molecular mass 882 Da. Ion 923 corresponds to a saponin monoacetylated which 924 Da. And, the ion 965 equivalent to saponin with two acetate groups, with 966 Da. The phytochemical analysis allowed us to consider these compounds as antifungal bioactive.

### 2.2. Tests of Susceptibility to Antifungals

We evaluated the following 125 yeasts that were obtained from vaginal samples: 60 *C. glabrata*, 30 *C. albicans*, 15 *C. tropicalis*, 11 *C. parapsilosis*, and nine *Saccharomyces cerevisae*. The minimum inhibitory concentration (MIC) ranges for HE, fluconazole, and nystatin are shown in Table 1. With regard to the *in vitro* susceptibility test for fluconazole, the majority of the isolates of *C. glabrata* were dose-dependent susceptible (DDS), some isolates were resistant, and a minority of isolates was susceptible. Of the nine isolates of *S. cerevisiae*, five were DDS and one was resistant to fluconazole. *C. tropicalis* and *C. parapsilosis* had only two isolates, and both were DDS. Among *C. tropicalis*, only one isolate was resistant. All of the *C. albicans* isolates were susceptible. For nystatin, of the 30 isolates of *C. albicans*, 14 were DDS. *C. glabrata* had only six isolates that were DDS. *C. tropicalis* had only one isolate that was DDS. For *C. parapsilosis* and *S. cerevisiae*, all of the isolates were susceptible to nystatin. For HE, all of the yeasts tested showed wide variations in MIC values. Considering the MIC50, we observed values that suggest good effectiveness, especially against CNCA.

**Table 1:** The minimum inhibitory concentration range and MIC 50 for HE fluconazole and nystatin against 125 vaginal yeasts including *Candida* and *Saccharomyces* genus.

	HE		Fluconazole		Nystatin	
	MIC Range	MIC50	MIC Range	MIC50	MIC Range	MIC50
	(µg/mL)					
*Candida albicans*	390–1560	1560	0.125–1	0.125	4–8	8
*Candida glabrata*	97.5–12,500	780	0.25–64	32	0.5–8	2
*Candida tropicalis*	780–3125	780	0.25–64	0.5	0.5–4	2
*Candida parapsilosis*	780–12,500	1560	1–16	2	2–4	2
*Saccharomyces cerevisae*	390–12,500	780	4–64	16	2–4	2

Note: MIC50: MIC for 50% of all isolates.

### 2.3. Time-Kill Assay

**Figure 2:**
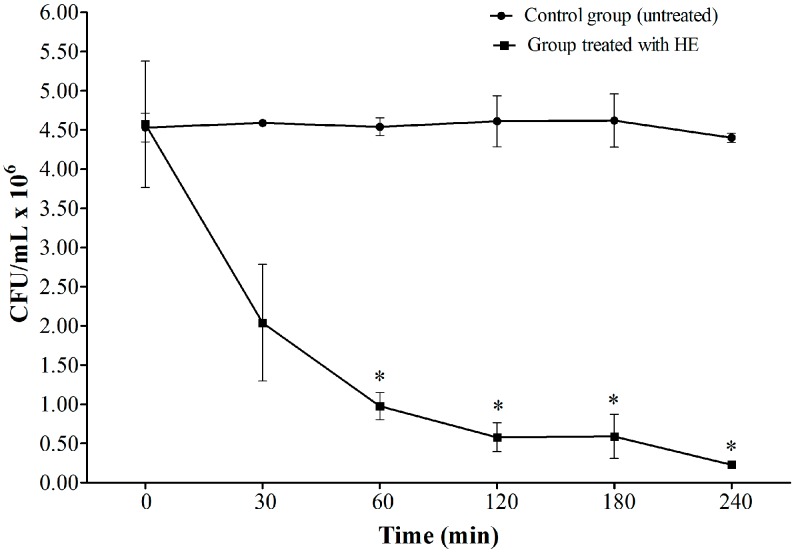
Time kill profile of *C. albicans* ATCC 90028 during exposition to HE (1560 µg/mL). CFU/mL: Colony forming unit per mililitre. * Indicates significant reduction in CFU.

Based on the results of the susceptibility tests, in which HE inhibited the growth of yeast, we determined the duration of this effect. A time-kill assay was performed to determine the growth profile curves for *C. albicans* ATCC 90028 in YPD broth that was exposed to 1560 µg/mL HE (*i.e*., the highest MIC value found) for 240 min (Figure 2). A substantial reduction of colony forming units (CFU) was observed compared with the control group. These data indicate the fungicide activity of HE in the first 60 min of exposure. After this time, we still observed a slight reduction of CFU until 240 min.

### 2.4. Scanning Electron Microscopy

**Figure 3:**
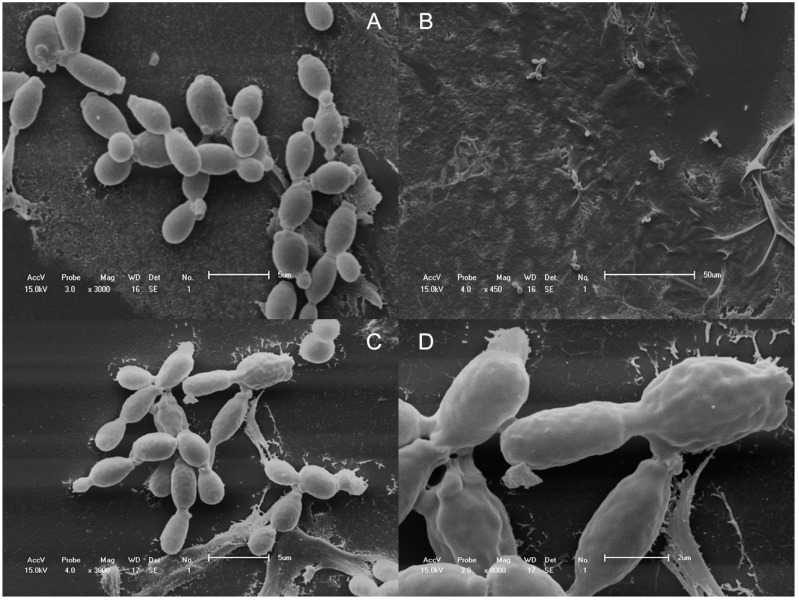
SEM micrographs of the untreated (**A**), 30 min (**B**) and 120 min (**C**,**D**) HE (1560 µg/mL) treated *C. albicans* ATCC 90028 cells.

Scanning electron microscopy (SEM) was used to observe surface alterations or general morphological changes in *C. albicans* ATCC 90028 cells after exposure to HE. Comparisons were made between untreated yeasts (control) and cells in the similar inoculum treated with 1560 µg/mL HE. All of the *C. albicans* cells in the control group generally had smooth-walled bodies, were spherical in shape, and were mostly present in the yeast form in large quantities (Figure 3A), after 30 min of exposure to HE, we observed a significant reduction of the amount of yeasts (Figure 3B). After 120 min, a few remaining yeasts had irregularities on their surface, such as convolutions (Figure 3C,D) which are not observed in Figure 3A.

### 2.5. Transmission Electron Microscopy

**Figure 4:**
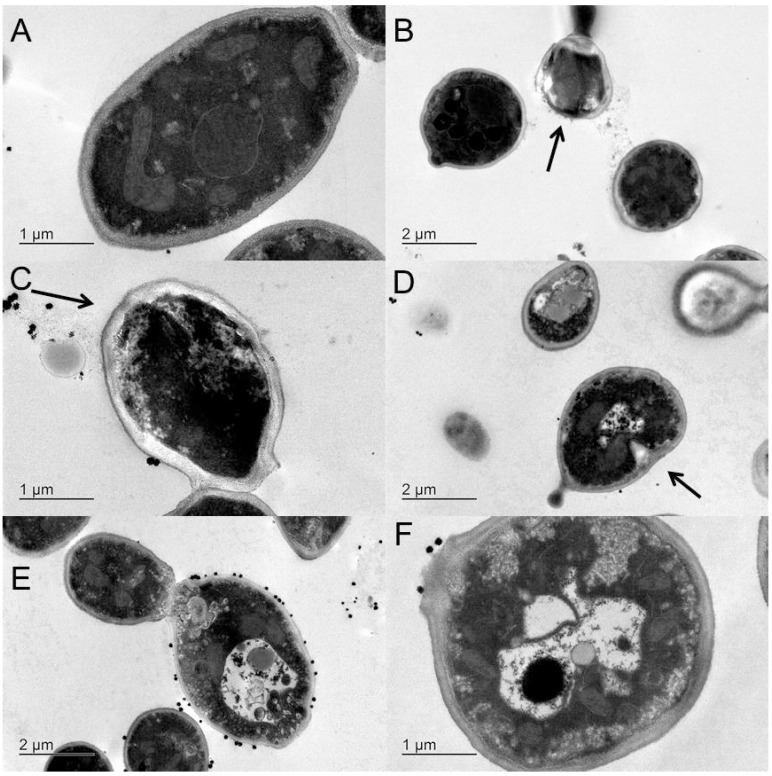
TEM micrographs of the untreated (**A**), 30 min (**B** and **C**) and 120 min (**D**–**F**) treated cells of *C. albicans* ATCC 90028 with HE with concentration of 1560 µg/mL.

Transmission electron microscopy (TEM) revealed that the untreated yeast cells in the control group had typical *C. albicans* morphology, with a uniform central density, structured nucleus, and cytoplasm with several elements of an endomembrane system that was enveloped by a regular, intact cell wall and plasma membrane that lied close to the cell wall (Figure 4A). After *C. albicans* cells were exposed to 1560 µg/mL HE for 30 min, we observed the presence of cell lysis, with loss of the cell wall (Figure 4B) and rupture of the cytoplasmic membrane, leading to the loss of intracellular material and cell wall irregularities (Figure 4C). After 120 min exposure to HE, some cells showed cytoplasmic membrane invaginations (Figure 4D) that caused marked structural disorganization within the cytoplasm and reduced intracellular volume (Figure 4E,F).

### 2.6. Discussion

In the present study, we confirmed the *in vitro* fungicide activity of HE from *S. saponaria* and observed some structural changes in *C. albicans* ATCC 90028 cells. We tested the antifungal activity of HE against yeasts from human vaginal samples, including four different species of the *Candida* genus and *S. cerevisae*. The data obtained for HE were compared with fluconazole and nystatin, two antifungal drugs currently used for VVC treatment. Our results initially confirmed the findings of Tsuzuki *et al.* [15], who reported the antifungal activity of a crude extract from *S. saponaria*. The present results are also consistent with our previous study [16] that reported the antifungal activity of this material. HE is a fraction of the extract from *S. saponaria* and is rich in two saponins: 3-*O*-(4-acetyl-β-d-xylopyranosyl)-(1→3)-α-l-rhamnopyranosyl-(1→2)-α-l-arabinopyranosyl-hederagenin and 3-*O*-(3,4-di-acetyl-β-d-xylopyranosyl)-(1→3)-α-l-rhamnopyranosyl-(1→2)-α-l-arabinopyranos-yl-hederagenin (S2) [15]. From fractionation of the precipitate, it was possible to isolate saponins S1 and S2.

Among the 125 yeasts that were evaluated in the present study, the median MIC (MIC50) was 780 µg/mL. Thus, according to the classification proposed by Duarte *et al.* [17] for the fungal inhibitory activity of plants, MIC values between 600 and 1500 μg/mL are considered as moderate inhibition. Our data are consistent with reports for another saponin, SC-2, obtained from *Solanum chrysotrichum*, which possesses fungistatic and fungicidal activity against *C. albicans* and other multi-resistant *Candida* species [18].

Importantly, we chose a variety of vaginal yeasts, including species that are difficult to treat, such as *C. glabrata.* This species responds poorly to single oral doses of 150 mg fluconazole [19] and exhibits intrinsically reduced susceptibility to azole drugs [20]. Our *in vitro* susceptibility results for fluconazole against *C. glabrata* are consistent with previous studies that demonstrated that vaginal isolates of CNCA, principally *C. glabrata*, are less susceptible to azoles than *C. albicans* [4,21]. These results that show *C. glabrata* resistance *in vitro* are important because there are few treatment options available for managing patients with VVC caused by these resistant yeasts [4,22]. Surprisingly, in the present study, specifically considering *C. glabrata*, the MIC50 was also 780 µg/mL. Therefore, this species did not require more concentration of HE, and its MIC50 value was identical to the one obtained for the whole collection of yeast. Our results confirm the antifungal activity of HE, suggesting that HE may be promising for VVC treatment. We previously reported the *in vitro* and *in vivo* activity of HE and a butanolic fraction [16] of an extract from *S. saponaria* against azole-resistant vaginal isolates of *C. glabrata* and azole-susceptible and -resistant isolates of *C. albicans* [16]. HE also showed excellent *in vitro* antifungal activity against the three main causal agents of VVC, including *C. albicans*, *C. glabrata*, and *C. tropicalis*.

Furthermore, *C. tropicalis* is also difficult to treat [23], but isolates of this species were inhibited *in vitro* by lower concentrations of HE compared with *C. albicans*, a species that is typically susceptible to fluconazole. Another important issue is that one isolate of *C. tropicalis* showed resistance to fluconazole (64 µg/mL) but was inhibited by HE at a median concentration of 780 µg/mL. Our results are encouraging because they demonstrate the action of HE against a wide range of clinical samples, many of which are resistant to fluconazole, thus confirming the effectiveness of HE.

Our enthusiasm for HE is reinforced by another vaginal application. Our research group recently found that HE as well as the butanolic extract from *S. saponaria* had anti-*Trichomonas vaginalis* activity *in vitro* [24]. The MIC for *T. vaginalis* was also 1560 µg/mL, which coincides with the maximum concentration required in the present study for *C. albicans*. Damke [24] reported that HE had no action against *Lactobacillus acidophilus* and thus may be a promising option for the treatment of VVC, and at the same time trichomoniasis without compromising the vaginal microbiota.

*C. albicans* is the species most frequently associated with VVC, and we found good action of HE against *C. albicans*. Moreover, in a previous study, our group observed the fungicide activity of HE. In the present study, we performed a time-kill assay to determine how much time is required for HE to begin exerting its fungicidal effects. The main components of HE observed in the present study were saponins. Knowing the amphoteric features of this chemical group, we hypothesized that these saponins bind components of the cytoplasmic membrane in yeast cells and cause cell lysis, which occurred within minutes after contact (Figure 2). After 30 min, we observed a sharp drop in CFU, which persisted up to 120 min and reached a plateau until 240 min (Figure 3B). A significant reduction of the amount of yeast was observed after 30 min exposure to HE. Figure 4B,C show rupture of the cell wall, indicating cell lysis. Over time, the interaction between saponins and the membrane constituents of yeast cells caused the loss of intracellular contents and cell wall disorganization, causing the presence of irregularities in the cell wall (Figure 3C) and consequently cell death.

The principal mechanism of the antifungal activity of saponins is their interaction with steroids of the fungal plasmatic cell membrane [25]. In this previous study, the authors mentioned that some plants contain saponins with proven antifungal activity, including *Kalopanax pinctus* against *C. albicans* and *Cryptococcus neoformans* and *Aspargus officinalis* against different types of fungi. These data indicate that plants that contain saponins are promising new antifungal therapies [26].

Phytochemical analyses of some species of the genus *Sapindus* have shown that they are rich in triterpenoid saponins and contain oleanoic acid and hederagenin with aglycones [27]. These compounds were also found in *S. saponaria* as saponins S1 and S2 [15], justifying our findings regarding damage to the yeast cell membrane. Our results are in agreement with Murgu and Rodrigues-Filho [13], who reported the presence of acyclic sesquiterpene oligoglycoside and saponins in young fruits of *S. saponaria* L.

In recent years, triterpenoid saponins, have been found to possess antifungal activity against *C. glabrata*, *C. albicans*, *Trichosporon beigelii*, *Penicillium avelaneum*, *Pyricularia oryzae*, *Cryptococcus neoformans*, *Coccidioides immitis*, *Saccharomyces cerevisiae*, and the dermatophytes *Microsporum canis* and *Trichophyton mentagrophytes* [28]. Saponins belong to oleane-type triterpenoid saponins, with C-28 and C-30 dicarboxylic groups and an olefinic double bond at C-12, which also showed antifungal activity against a panel of human-pathogenic opportunistic fungi [29]. Recently, Herrera-Arellano [18] reported that saponin SC-2 from *Solanum chrysotrichum* caused morphological changes and cell death in *C. albicans*. These authors also considered that SC-2 might be an alternative therapy for the treatment of VVC. We believe the antifungal activity of HE also may be attributable to the saponins present in its composition.

The present results and our previous study indicate that HE has important antifungal activity both *in vitro* and *in vivo* and appears to be a promising alternative for the treatment of patients with VVC. Low concentrations eliminated the infection in azole-resistant vaginal isolates of *C. glabrata* and azole-susceptible and -resistant isolates of *C. albicans* taken from experimental rats. We previously found that HE is not cytotoxic in cell cultures. These results reinforce the possibility that HE may substitute for traditional drugs in the treatment of VVC. The treatment options for VVC are still very limited. The polyene derivatives nystatin and amphotericin B are the only presently available fungicidal drugs, but these drugs present toxicity, and some isolates were DDS or resistant to classical antifungals. Fluconazole is the most frequently used antifungal for the treatment of VVC, but it is only fungistatic and presents poor activity against CNCA isolates.

## 3. Experimental Section

### 3.1. Preparation of HE

HE was obtained according a previous study [15]. Fruits of *S. saponaria* were collected on the campus of the State University of Maringá, Paraná, Brazil (UEM). The plant was identified by staff members in Department of Botany at UEM. An exsiccate was deposited in the Herbarium of UEM (HUM 11710). The dried pericarps of the fruits (450.0 g) of *S. saponaria* were ground and extracted with EtOH:H_2_O (v/v, 9:1,) at room temperature through a process of dynamic maceration with constant mechanical stirring. Extraction was performed in an amber flask, maintained at ambient temperature for 6 consecutive days (6 h/day). The extract was concentrated under low pressure in a rotary evaporator at 40 °C. After elimination of the solvent, the extract was frozen in liquid nitrogen and lyophilized in a Alpha 1–2 freeze dryer Martin Christ (Osterode am Harz, Niedersachsen, Germany). The lyophilized extract was stored in a closed plastic flask and kept frozen. The HE stock was diluted in Milli-Q water at 25 mg/mL.

### 3.2. Characterization of HE

The nuclear magnetic resonance spectra were obtained in a Varian Gemini 97.05T using deuterated solvent and TMS as the internal standard at a constant temperature of 298K. IR: film NaCl plates; ESI-MS were recorded on a Micromass Quattro LC, HRMS: CC: silica gel 60 (70–230 and 230–400 mesh); TLC: silica gel plates F_254_ (0.25 mm thickness).

### 3.3. Samples

For the susceptibility testing of antifungals, we used yeast isolates from vaginal secretions from women that attended at the Teaching and Research in Clinical Analyses Laboratory (LEPAC) of UEM. The isolates were identified as *C. glabrata*, *C. albicans*, *C. tropicalis*, *C. parapsilosis*, and *S. cerevisae* in cervical-vaginal material collected for fungus culture. The samples were seeded on plates with Sabouraud Dextrose Agar (SDA; Difco, Sparks, MD, USA) plus 0.2 g/L chloramphenicol (Sigma, St. Louis, MO, USA) and incubated at 35 °C for 48–72 h. A pool of growing colonies was subcultivated in CHROMagar *Candida* (CHROMagar Company, Paris, France) to investigate the purity of the culture and colony’s color. Yeasts that grew in this differential selective medium were identified according to classic methodology [30]. A reference yeast, *C. albicans* (ATCC 90028), was added to all of the tests.

### 3.4. Tests of Susceptibility to Antifungals

The antifungal activity of HE was compared with the *in vitro* action of fluconazole and nystatin. Fluconazole and nystatin were used in powdered form prepared at 10 concentrations, varying from 0.125 to 64 μg/mL. The MICs for fluconazole, nystatin, and HE were determined by the broth microdilution method following recommendations of the Clinical and Laboratory Standards Institute (document no. M27-A3) [31]. HE was prepared in 10 concentrations, varying from 24.4 to 12,500 µg/mL. This variation was based on previous tests.

Susceptibility tests were performed after reactivating the yeasts in Sabouraud Dextrose Broth (SDB; Difco) for subsequent culture in SDA for 24 or 48 h at 30 °C. This growth was used to prepare an inoculum in sterile saline. The cell density was adjusted with the aid of a spectrophotometer at 530 nm with 90% ± 2% transmittance. This turbidity resulted in 1.0×5.0 × 10^6^ CFU/mL, which was used to prepare further dilutions in RPMI to obtain the desired final inoculum that contained 0.5–2.5 × 10^3^ CFU/mL. The microplates were incubated in a humidified chamber at 35 °C for 48 h. The test was performed in triplicate on three different days. The results refer to the mean of the values obtained. Following incubation, readings were performed in a microplate reader (Expert Plus, ASYS, Cambridge, UK).

The MIC was defined as the lowest concentration of the antifungal agent that is capable of causing 50% inhibition for fluconazole and 100% inhibition for nystatin (polyene) [31] and HE [16]. The results are expressed as susceptible (S), DDS, and resistant (R). The MIC50 was defined as the concentration that was capable of inhibiting 50% of the population of yeast.

### 3.5. Time-Kill Assay of C. albicans against HE

For this assay, the standard strain of *C. albicans*, ATCC 90028, was reactivated in 2 mL SDB and maintained in an environmental chamber for 48 h. Isolated colonies were then obtained on SDA after 24 h at 37 °C. Some colonies were transferred to 20 mL of YPD (1% yeast extract, 2% peptone, and 2% dextrose), and the culture was maintained under stirring overnight at 37 °C.

The optical density (OD) of the culture was checked by reading absorbance in a spectrophotometer. The culture was diluted with YPD to achieve an OD of 0.1, with a cell density of 10^4^ CFU/mL. Cell counting was performed in a Neubauer chamber, and the number of cells was approximately 5 × 10^4^ CFU/mL. The culture was again maintained under stirring at 37 °C for approximately 5 h until the number of cells reached approximately 5 × 10^6^ CFU/mL, indicating the logarithmic phase of yeast growth.

After this time, cell counting was performed again, and cells were distributed into individual culture flasks that contained HE at a concentration of 1560 µg/mL (equivalent to MIC). After the addition of drugs, plating was performed to determine CFU counts at 0, 15, 30, 60, 120, 180, and 240 min. At each of these time intervals, aliquots were taken for SEM and TEM. For plating, dilutions of the samples were made, and a positive control (yeast without drug exposure) was added for comparisons with the growth of the yeasts. Plating was performed on SDA cultures and maintained for 24 h at 35 °C. The number of colonies was determined for each plate count.

### 3.6. Scanning Electron Microscopy

The aliquots that were taken at 0, 15, 30, 60, and 120 min were washed, fixed in a solution of 2.5% glutaraldehyde in 0.1 M cacodylate buffer (Sigma), and dehydrated in an ascending series of alcohol. Critical-point drying was performed using a Balzers CPD-010 (Balzers Instruments, Balzers, Liechtenstein) with carbonic gas. Metallization in gold was performed using a Balzers SCD-030 (Balzers Instruments). The samples were observed and photographed with a JEOL-JSM 6360 LV scanning electron microscope (JEOL Ltd., Tokyo, Japan) at the Electron Microscopy Center, Federal University of Paraná.

### 3.7. Transmission Electron Microscopy

The aliquots that were taken at 0, 15, 30, 60, and 120 min were washed, fixed in a solution of 2.5% glutaraldehyde in 0.1 M cacodylate buffer (Sigma), and then washed three times for 10 min in 0.1 M cacodylate buffer, pH 7.2, at 48 °C. The material was then post-fixed in 2.0% osmium tetroxide in 0.1 M cacodylate buffer, pH 7.2, for 1 h. En bloc staining was done with 2.0% uranyl acetate for 2 h. The material was dehydrated in an ascending series of alcohol and then placed in acetone. Infiltration and embedding were performed in Epon-812 resin (EMBed-812 Embedding Kit, catalog no. 14120, Electron Microscopy Sciences, Hatfield, PA, USA). Sections were cut with a Porter Blum MT-2 ultramicrotome (Sorval, Liverpool, NY, USA) using glass and diamond blades. Ultrathin sections were contrasted in an aqueous solution of 2.0% uranyl acetate and lead nitrate/acetate. Material from all of the samples was observed under a JEOL 1200EX II transmission electron microscope at the Electron Microscopy Center, Federal University of Paraná.

### 3.8. Statistical Analysis

Data were expressed as the mean ± standard deviation (SD) of at least three independent experiments. Significant differences among means were identified using analysis of variance (ANOVA) followed by the Bonferroni test. The data were analyzed using Prism 6.0 software (GraphPad, San Diego, CA, USA). Values of *p* ≤ 0.05 were considered statistically significant.

## 4. Conclusions

In the present study, we confirmed the *in vitro* fungicide activity of hydroalcoholic extract from *S. saponaria*, by evaluation of 125 vaginal yeasts including *Candida* and *Saccharomyces* genus. Some isolates required high concentrations of extract, but the majority of the MIC values suggest good antifungal effectiveness, especially against CNCA. In addition it was proved for the first time that the fungicide activity of HE occurs quickly, within the first 60 min of exposure. Moreover it was possible observed some important structural changes in *C. albicans* cells after exposition to HE, such as cell lysis, loss of the cell wall, rupture of the cytoplasmic membrane, loss of intracellular material and cell wall irregularities. A prolonged exposure to HE (120 min) caused cytoplasmic membrane invaginations with marked structural disorganization of cytoplasm and reduced intracellular volume. Our results so far allow us consider HE a promising alternative for the management of candidiasis, which could contribute to the pharmaceutical treatment arsenal, especially of VVC. However, it is possible that purified components may have even more potency with respect inhibition of yeasts. Thus, purified active principles should be further tested to determine their efficacy and toxicity and possible incorporation in pharmaceutical formulations to perform *in vivo* tests.

## References

[1] Sobel J.D. (2007). Vulvovaginal candidosis. Lancet.

[2] Achkar J.M., Fries B.C. (2010). *Candida* infections of the genitourinary tract. Clin. Microbiol. Rev..

[3] Workowski K.A., Berman S.M. (2010). Centers for disease control and prevention. Sexually Transmitted Diseases Treatment Guidelines 2010.

[4] Dalben Dota K.F., Shinobu C.S., Patussi E.V., Lopes Consolaro M.E., Estivalet Svidzinski T.I. (2008). Susceptibilidad de levaduras vaginales a los antifúngicos más utilizados en Maringá, Paraná, Brasil. Acta Bioquím. Clin. Latinoam..

[5] Ragunathan L., Poongothai G.K., Sinazer A.R., Kannaiyan K., Gurumurthy H., Jaget N., Kuthalaramalingam S. (2014). Phenotypic characterization and antifungal susceptibility pattern to fluconazole in *Candida* species isolated from vulvovaginal candidiasis in a tertiary care hospital. J. Clin. Diagn. Res..

[6] Kumari V., Banerjee T., Kumar P., Pandey S., Tilak R. (2013). Emergence of non-*albicans*
*Candida* among candidal vulvovaginitis cases and study of their potential virulence factors, from a tertiary care center, North India. Indian J. Pathol. Microbiol..

[7] Liu X.P., Fan S.R., Peng Y.T., Zhang H.P. (2014). Species distribution and susceptibility of *Candida* isolates from patient with vulvovaginal candidiasis in Southern China from 2003 to 2012. J. Med. Mycol..

[8] Espinel-Ingroff A., Canton E. (2008). Comparison of neo-sensitabs tablet diffusion assay with CLSI broth microdilution M38-A and disk diffusion methods for testing susceptibility of filamentous fungi with amphotericin B, caspofungin, itraconazole, posaconazole, and voriconazole. J. Clin. Microbiol..

[9] Ghannoum M.A., Rice L.B. (1999). Antifungal agents: Mode of action, mechanisms of resistance, and correlation of these mechanisms with bacterial resistance. Clin. Microbiol. Rev..

[10] Iavazzo C., Gkegkes I.D., Zarkada I.M., Falagas M.E. (2011). Boric acid for recurrent vulvovaginal candidiasis: The clinical evidence. J. Women’s Health.

[11] Dota K.F.D., Consolaro M.E.L., Svidzinski T.I.E., Bruschi M.L. (2011). Antifungal activity of brazilian propolis microparticles against yeasts isolated from vulvovaginal candidiasis. Evid. -Based Complement. Altern. Med..

[12] Meyer Albiero A.L., Aboin Sertié J.A., Bacchi E.M. (2002). Antiulcer activity of *Sapindus saponaria* L. in the rat. J. Ethnopharmacol..

[13] Murgu M., Rodrigues-Filho E. (2006). Dereplication of glycosides from *Sapindus saponaria* using liquid chromatography-mass spectrometry. J. Braz. Chem. Soc..

[14] Rashed K.N., Ćirić A., Glamočlija J., Calhelha R.C., Ferreira I.C.F.R., Soković M. (2013). Antimicrobial activity, growth inhibition of human tumour cell lines, and phytochemical characterization of the hydromethanolic extract obtained from *Sapindus saponaria* L. aerial parts. BioMed Res. Int..

[15] Tsuzuki J.K., Svidzinski T.I., Shinobu C.S., Silva L.F., Rodrigues-Filho E., Cortez D.A., Ferreira I.C. (2007). Antifungal activity of the extracts and saponins from *Sapindus saponaria* L.. An. Acad. Bras. Cienc..

[16] Damke E., Tsuzuki J.K., Cortez D.A., Ferreira I.C., Bertoni T.A., Batista M.R., Donati L., Svidzinski T.I., Consolaro M.E. (2011). *In vivo* activity of *Sapindus saponaria* against azole-susceptible and resistant human vaginal *Candida* species. BMC Complement. Altern. Med..

[17] Duarte M.C.T., Figueira G.M., Sartoratto A., Rehder V.L.G., Delarmelina C. (2005). Anti-*Candida* activity of brazilian medicinal plants. J. Ethnopharmacol..

[18] Herrera-Arellano A., López-Villegas E.O., Rodríguez-Tovar A.V., Zamilpa A., Jiménez-Ferrer E., Tortoriello J., Martínez-Rivera M.A. (2013). Use of antifungal saponin SC-2 of *Solanum chrysotrichum* for the treatment of vulvovaginal candidiasis: *In vitro* studies and clinical experiences. Afr. J. Tradit. Complement. Altern. Med..

[19] Goswami D., Goswami R., Banerjee U., Dadhwal V., Miglani S., Lattif A.A., Kochupillai N. (2006). Pattern of *Candida* species isolated from patients with *diabetes mellitus* and vulvovaginal candidiasis and their response to single dose oral fluconazole therapy. J. Infect..

[20] Vermitsky J.-P., Edlind T.D. (2004). Azole resistance in *Candida glabrata*: Coordinate upregulation of multidrug transporters and evidence for a PDR1-like transcription factor. Antimicrob. Agents Chemother..

[21] Ferrer J. (2000). Vaginal candidosis: Epidemiological and etiological factors. Int. J. Gynecol. Obstet..

[22] Dalben-Dota K.F., Faria M.G.I., Bruschi M.L., Pelloso S.M., Lopes-Consolaro M.E., Svidzinski T.I.E. (2010). Antifungal activity of propolis extract against yeasts isolated from vaginal exudates. J. Altern. Complement. Med..

[23] Barchiesi F., Calabrese D., Sanglard D., di Francesco L.F., Caselli F., Giannini D., Giacometti A., Gavaudan S., Scalise G. (2000). Experimental induction of fluconazole resistance in *Candida tropicalis* ATCC 750. Antimicrob. Agents Chemother..

[24] Damke E., Tsuzuki J.K., Chassot F., Cortez D.A.G., Ferreira I.C.P., Mesquita C.S.S., da-Silva V.R.S., Svidzinski T.I.E., Consolaro M.E.L. (2013). Spermicidal and anti-*Trichomonas vaginalis* activity of brazilian *Sapindus saponaria*. BMC Complement. Altern. Med..

[25] Francis G., Kerem Z., Makkar H.P.S., Becker K. (2002). The biological action of saponins in animal systems: A review. Br. J. Nutr..

[26] Negri M., Salci T.P., Shinobu-Mesquita C.S., Capoci I.R.G., Svidzinski T.I.E., Kioshima E.S. (2014). Early state research on antifungal natural products. Molecules.

[27] Murgu M., Santos L.F.A., Souza G.D.D., Daolio C., Schneider B., Ferreira A.G., Rodrigues-Filho E. (2008). Hydroxylation of a hederagenin derived saponin by a *Xylareaceous*
*fungus* found in fruits of *Sapindus saponaria*. J. Braz. Chem. Soc..

[28] Du Z., Zhu N., Ze-Ren-Wang-Mu N., Shen Y. (2003). Two new antifungal saponins from the tibetan herbal medicine *Clematis tangutica*. Planta Medica.

[29] Escalante A.M., Santecchia C.B., López S.N., Gattuso M.A., Gutiérrez Ravelo A., Delle Monache F., Gonzalez Sierra M., Zacchino S.A. (2002). Isolation of antifungal saponins from *Phytolacca tetramera*, an argentinean species in critic risk. J. Ethnopharmacol..

[30] Larone D.H. (2011). Medically Important Fungi: A Guide to Identification.

[31] Clinical and Laboratory Standards Institute (CLSI) (2008). Reference Method for Broth Dilution Antifungal Susceptibility Testing of Yeasts.

